# Supplementary material for: Short-chain ketone production by engineered polyketide synthases in Streptomyces albus
Source: Nat Commun. 2018 Nov 1;9:4569. doi: 10.1038/s41467-018-07040-0 (PMC6212451; doi:10.1038/s41467-018-07040-0)
Supplement: Supplementary file 1 — Supplementary Information [file 41467_2018_7040_MOESM1_ESM.docx]

**Short-chain ketone production by engineered polyketide synthases in *Streptomyces albus***

Yuzawa *et al.*

**
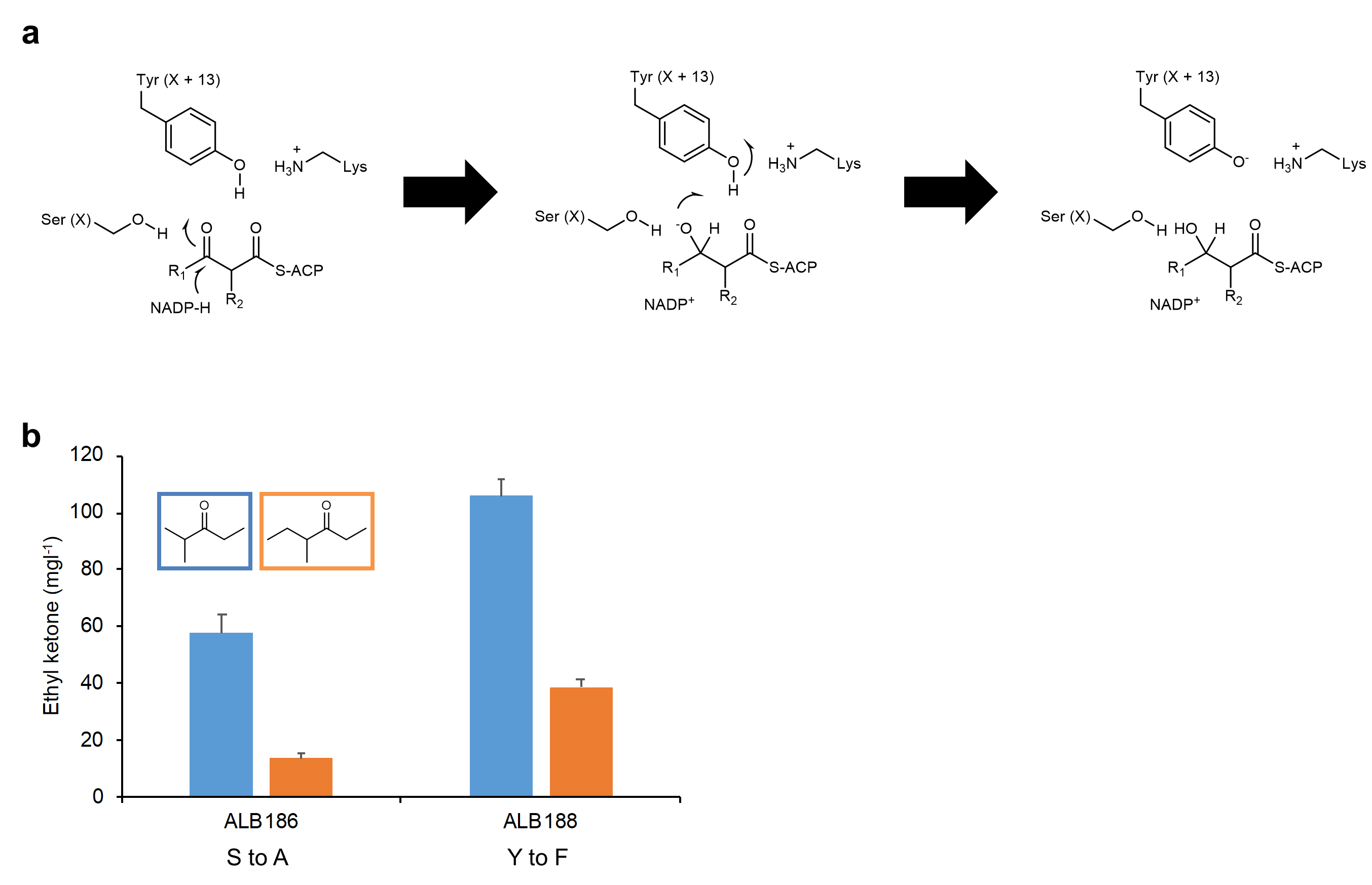
**

**Supplementary Fig. 1** **Two different KR inactivation strategies.** **a** Reduction catalyzed by a KR domain. A newly generated β-keto polyketide intermediate binds in the active site preloaded with NADPH. The β-carbonyl group activated by the Ser and Tyr residues is attacked by the NADPH hydride. The oxygen accepts a proton from the Tyr residue. To inactivate the KR, we mutated S1887 to A (ALB186) and Y1900 to F (ALB188). **b** Short-chain ketone production in shake flasks. ALB186 and ALB188 were grown in shake flasks containing 30 ml of Medium 042 at 30°C. Samples were taken after 5 days and production of 2-methyl-3-pentanone and 4-methyl-3-hexanone were measured by LC-MS. 2-Methyl-3-pentanone and 4-methyl-3-hexanone are shown in blue and orange, respectively. Error bars are the S.D. from three independent experiments. Abbreviations: ALB, *albus*; KR, ketoreductase.

**
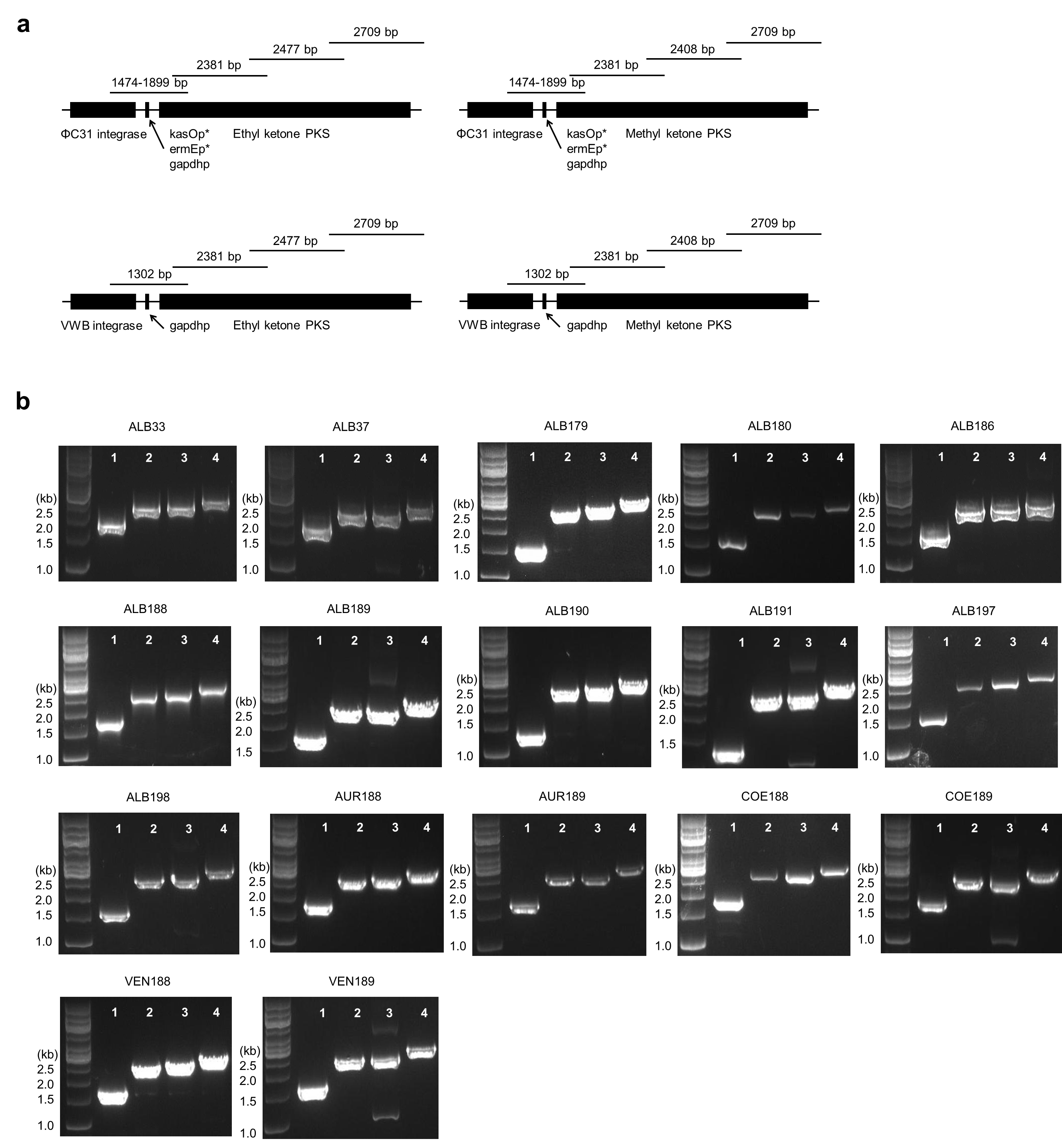
**

**Supplementary Fig. 2** **Genomic PCR analysis of engineered *Streptomyces* strains.** **a** Expected size of PCR fragments from each construct (see also Supplementary Table 3). **b** Genomic PCR data for *Streptomyces* strains that encode PKS genes. Lane 1 = the first PCR fragment (= 1302, 1474, 1497, 1662, or 1899 bp); Lane 2 = the second PCR fragment (= 2381 bp); Lane 3 = the third PCR fragment (= 2408 or 2477 bp); Lane 4 = the fourth PCR fragment (= 2709 bp). Abbreviations: AUR, *aureofaciens*; COE, *coelicolor*; PKS, polyketide synthase; VEN, *venezuelae*.

**
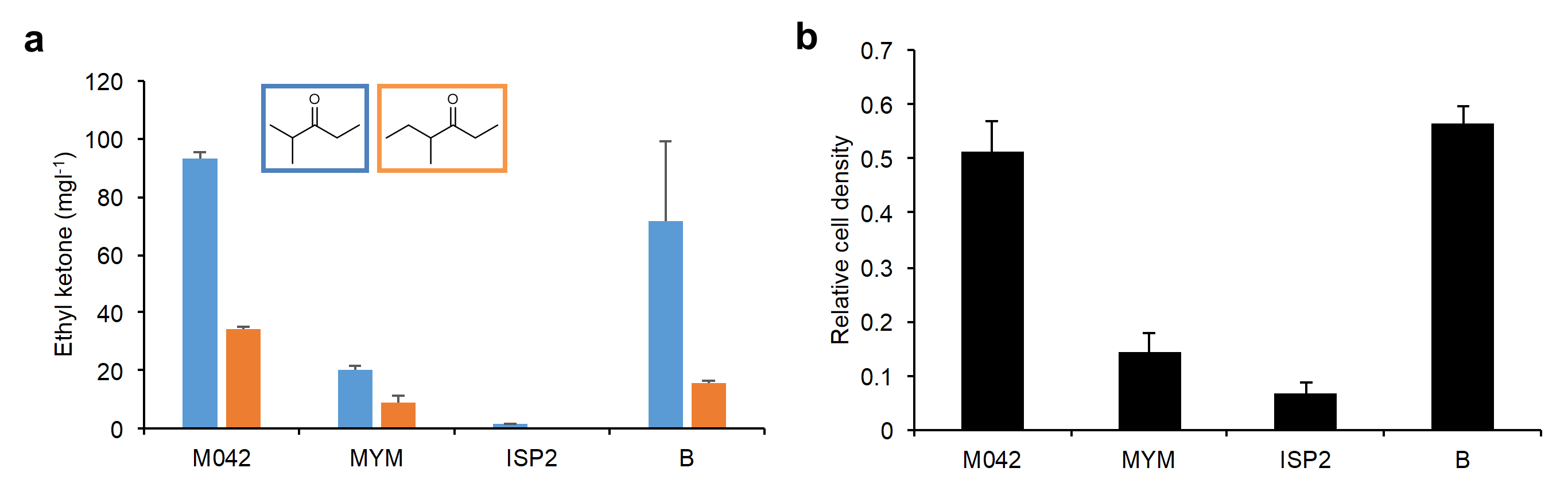
**

**Supplementary Fig. 3** **Ethyl ketone production by ALB188 grown in different media.** **a** ALB188 was cultured in M042, MYM, ISP2, or B for 5 days at 30°C and ethyl ketone production was measured by LC-TOF-MS (n = 3). 4-methyl-3-hexanone was quantified as 5-methyl-3-hexanone equivalent because 4-methyl-3-hexanone is not commercially available. 2-Methyl-3-pentanone and 4-methyl-3-hexanone are shown in blue and orange, respectively. Error bars are the S.D. from three independent experiments. **b** Relative cell density of ALB188 grown in M042, MYM, ISP2, or B at 5 days were estimated by the Bradford assay. Error bars are the S.D. from three independent experiments.


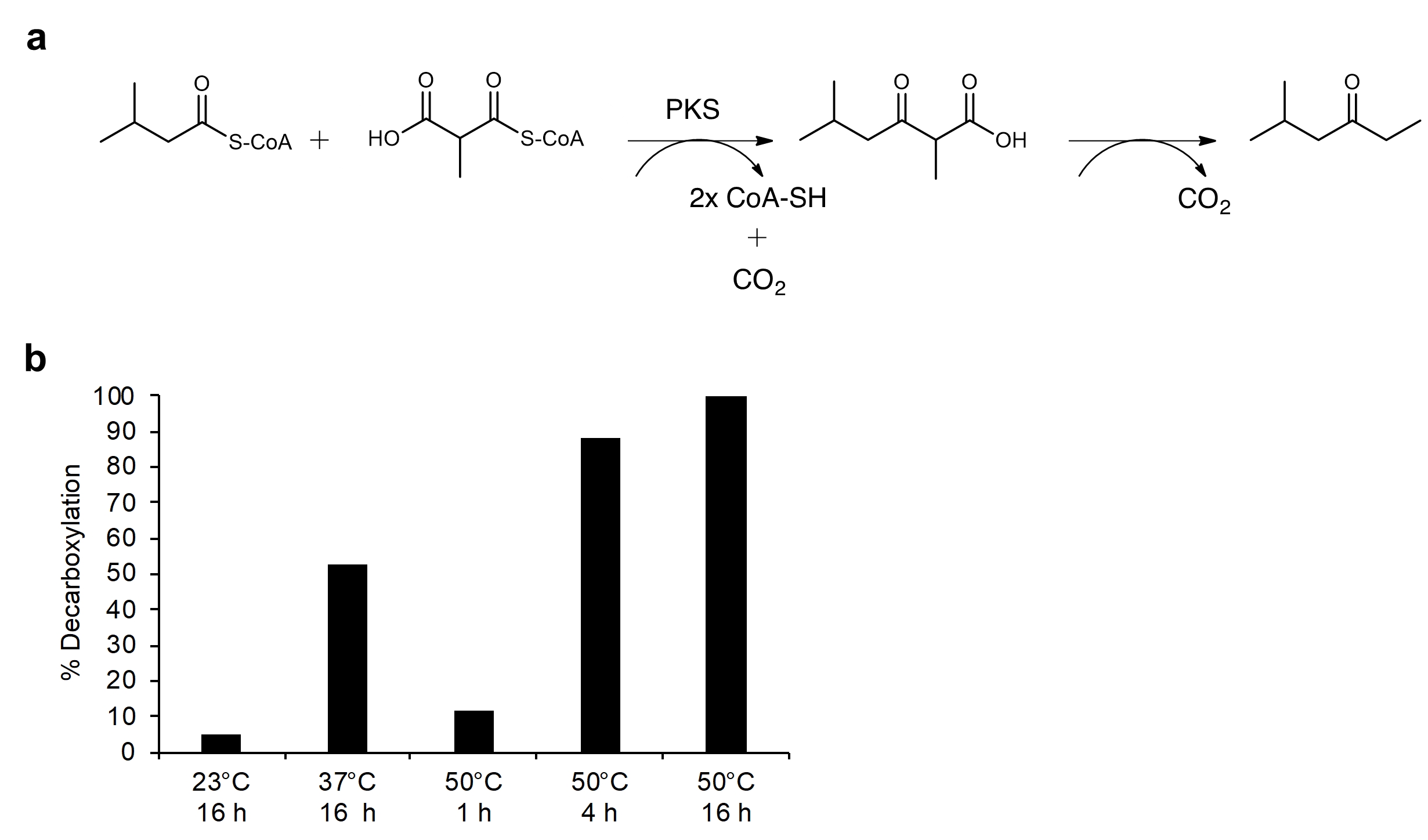


**Supplementary Fig. 4** **Decarboxylation of a model 3-keto acid *in vitro* and evaporation rates of short-chain ketones in shake flask conditions.** **a** 2,5-dimethyl-3-ketohexanoic acid was produced by LipPks1+TE from isovaleryl-CoA and methylmalonyl-CoA in the absence of NADPH *in vitro*. **b** The product was then incubated at different temperatures for different times and 5-methy-3-hexanone production was measured by LC-MS.


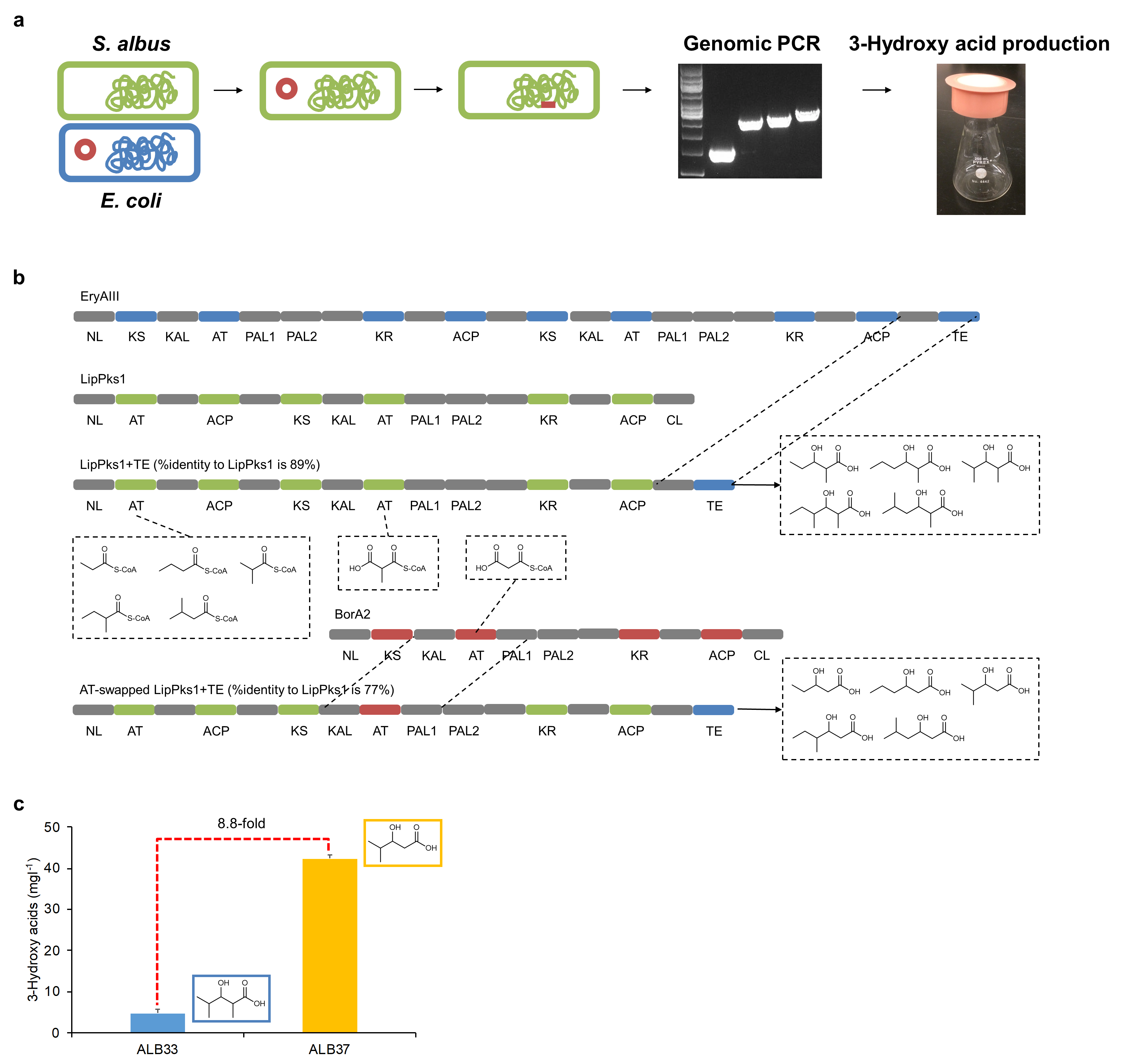


**Supplementary Fig. 5** **Hybrid PKSs used for 3-hydroxy acid production.** **a** Scheme of experimental procedures to engineer *Streptomyces* strains. Plasmids (red circle) carrying desired genes were constructed and introduced *into E. coli* and transferred to *S. albus* hosts via conjugation. Site-specific integration was carried out by the ΦC31 *attP* and integrase carried on the plasmid. Correct integration was confirmed by PCR. Colonies were picked and cultured for 3-hydroxy acid production. **b** PKS engineering strategy. Catalytic domains of LipPks1, EryAIII, and BorA2, are shown in green, blue, and red, respectively. Linkers and subdomains in each PKS are shown in gray. BLAST Global align was used to calculate sequence identity of each hybrid enzyme. Expected compounds produced by LipPks1+TE and the AT-swapped version are shown in dashed boxes after arrows. Acyl-CoAs and carboxyacyl-CoAs shown are specified by the AT domain shown by the dotted line. The sequence identity of each synthetic enzyme to its parent enzyme was calculated using BLAST Global Align (https://blast.ncbi.nlm.nih.gov/Blast.cgi). **c** 3-Hydroxy acid production using *S. albus* engineered with two different hybrid PKSs*.* ALB33 and ALB37 that encode LipPks1+TE and AT-swapped LipPks1+TE, respectively, were cultured in Medium 042 for 5 days at 30°C. 3-Hydroxy acid production was measured by LC-MS. 2-Methyl-3-pentanone and 4-methyl-3-hexanone are shown in blue and yellow, respectively. Error bars are the S.D. from three independent experiments. Abbreviations: AT, acyltransferase; ACP, acyl carrier protein; CoA, coenzyme A; CL, *C*-terminal linker; KAL, KS to AT linker; KS, ketosynthetase; NL, *N*-terminal linker; PAL, Post AT linker; TE, thioesterase.

**
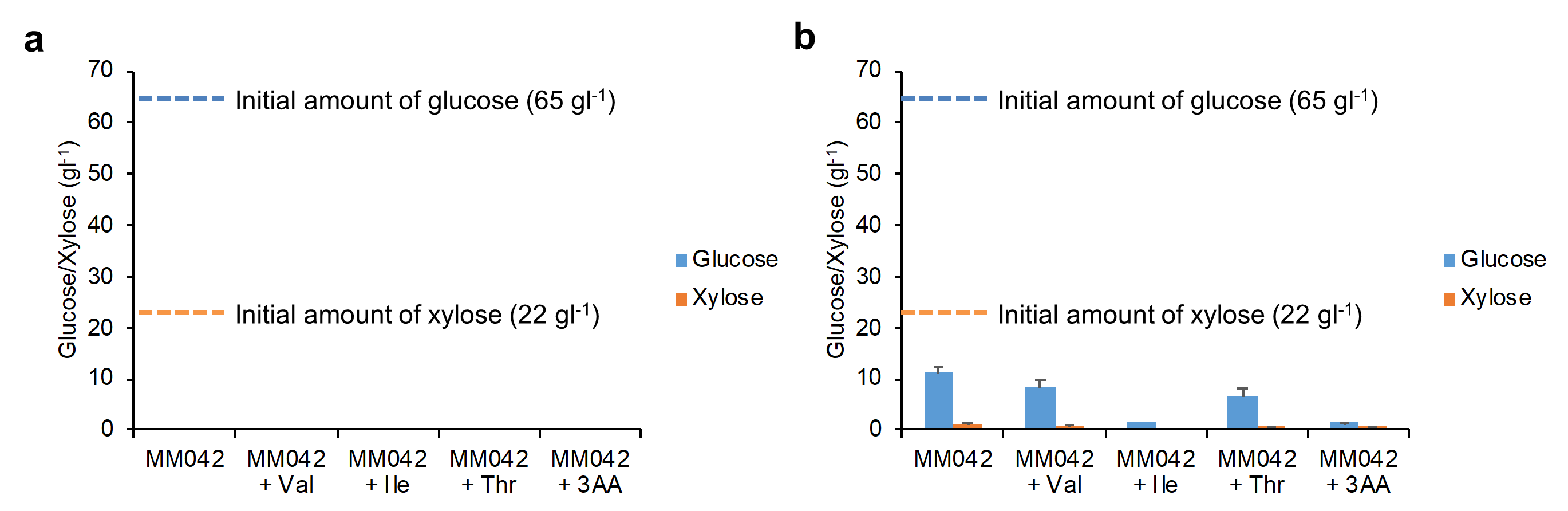
**

**Supplementary Fig. 6** **Sugar consumption analysis.** After culturing ALB188 (**a**) and ALB191 (**b**) in MM042 or MM042 + amino acids (Val, Ile, The, or the 3 amino acids) for 9 days, amounts of glucose and xylose were measured by LC-MS. Error bars are the S.D. from three independent experiments. Remaining amounts of glucose and xylose in (**a**) were <0.16 g⋅l^-1^ and <0.1 g⋅l^-1^, respectively.

**
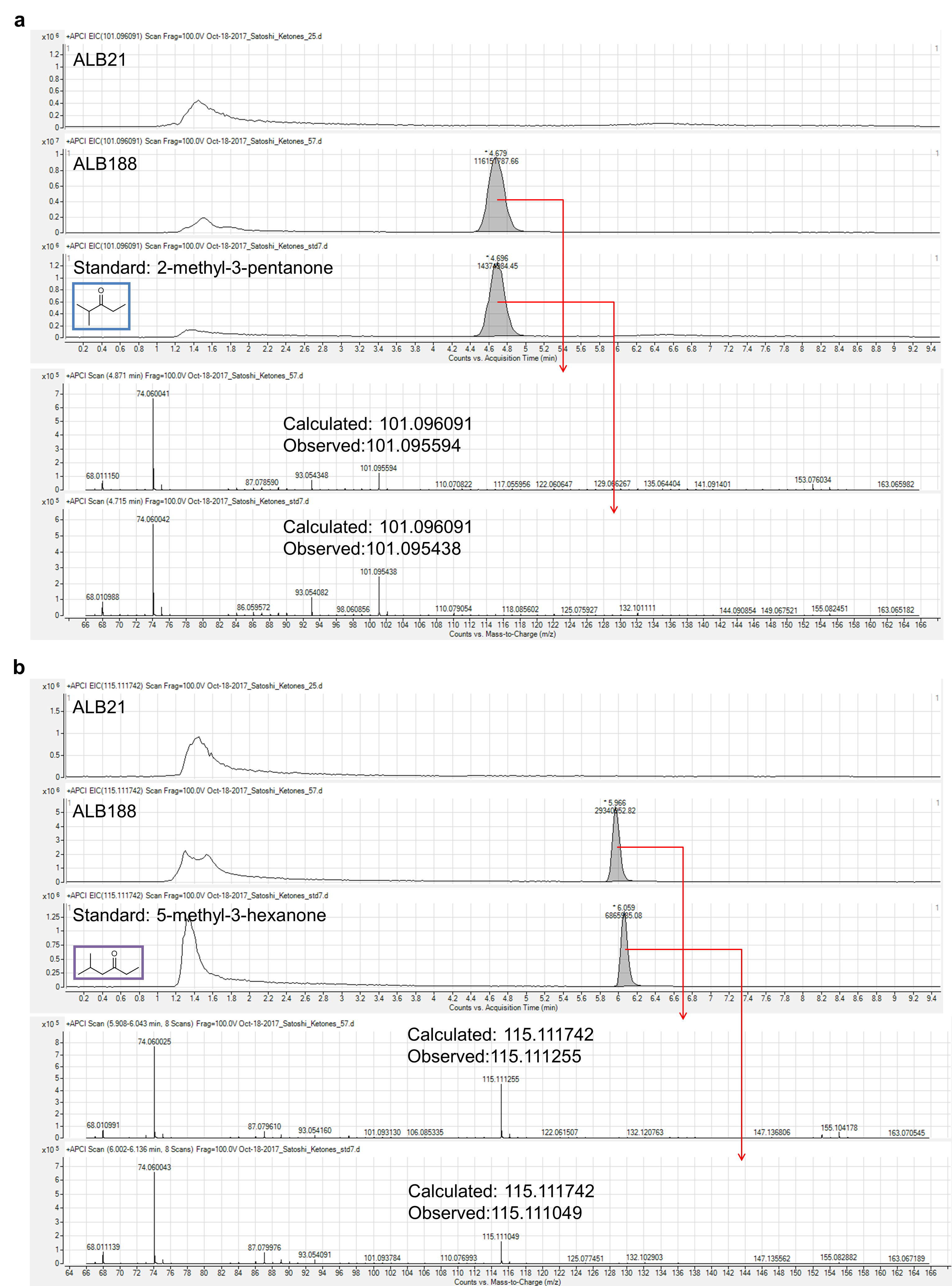
**

**Supplementary Fig. 7** **Representative LC chromatograms and MS data for ketone production.** ALB21 and ALB188 were grown in MM042 for 9 days and ketone productions were analyzed by LC-TOF-MS. **a** Extracted ion chromatograms at 101.096091 for ALB21 and ALB188 and the corresponding MS data. **b** Extracted ion chromatograms at 115.111742 for ALB21 and ALB188 and the corresponding MS data. 5-methyl-3-hexanone was used as a standard because 4-methyl-3-hexanone is not commercially available.


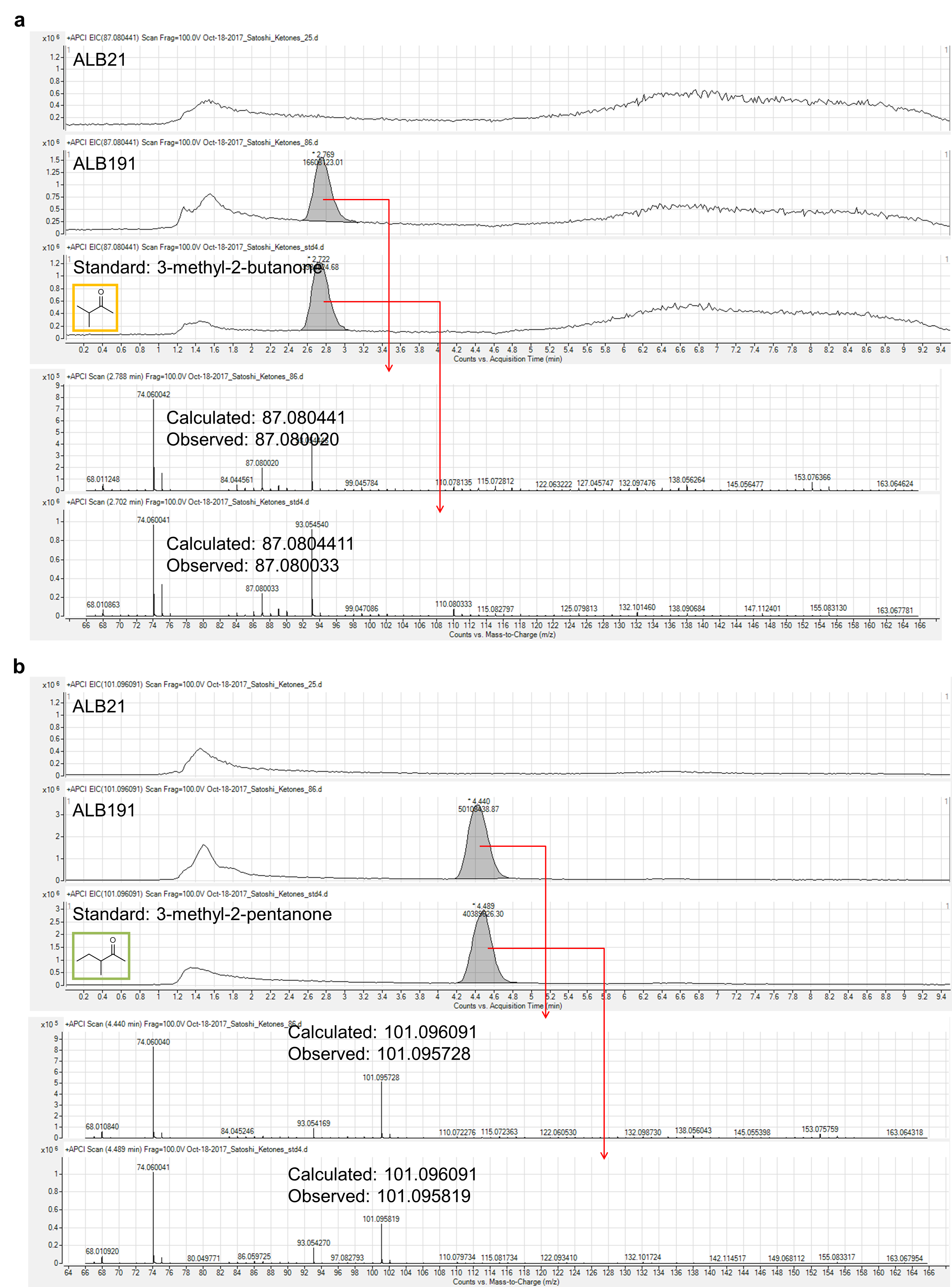


**Supplementary Fig. 8** **Representative LC chromatograms and MS data for ketone production**. ALB21 and ALB191 were grown in MM042 for 9 days and ketone productions were analyzed by LC-TOF-MS. **a** Extracted ion chromatograms at 87.0804411 for ALB21 and ALB191 and the corresponding MS data. **b** Extracted ion chromatograms at 101.096091 for ALB21 and ALB191 and the corresponding MS data.


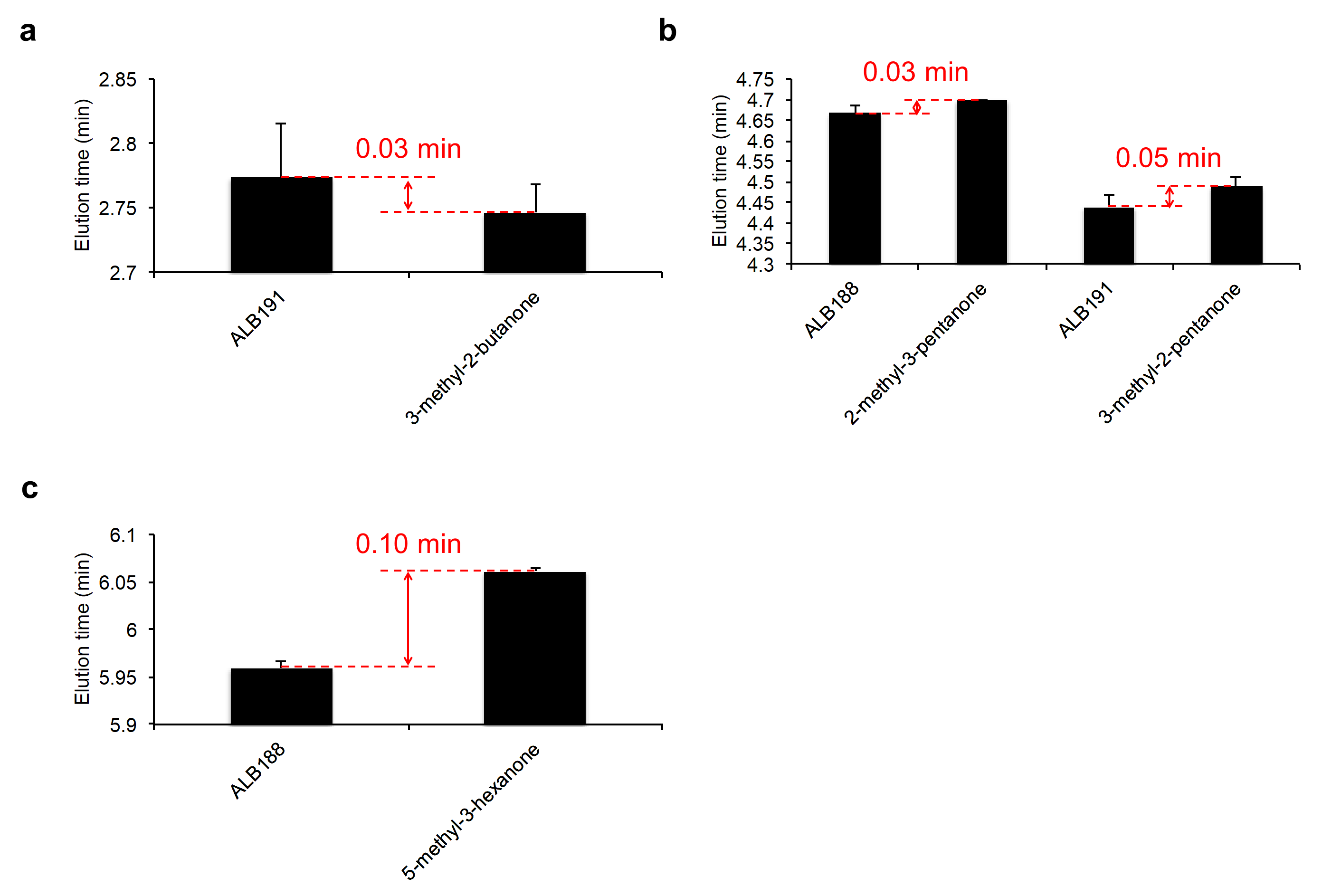


**Supplementary Fig. 9** **Elution time difference between ketone products and the corresponding standards used.** Error bars are the S.D. from three independent experiments. **a** Extracted ion chromatograms at 87.0804411 for ALB191 and 3-methyl-2-butanone standard were compared. **b** Extracted ion chromatograms at 101.096091 for ALB188, ALB191, 2-methyl-3-pentanone standard, and 3-methyl-2-pentanone standard were compared. **c** Extracted ion chromatograms at 115.111742 for ALB188 and 5-methyl-3-hexanone standard were compared.

**
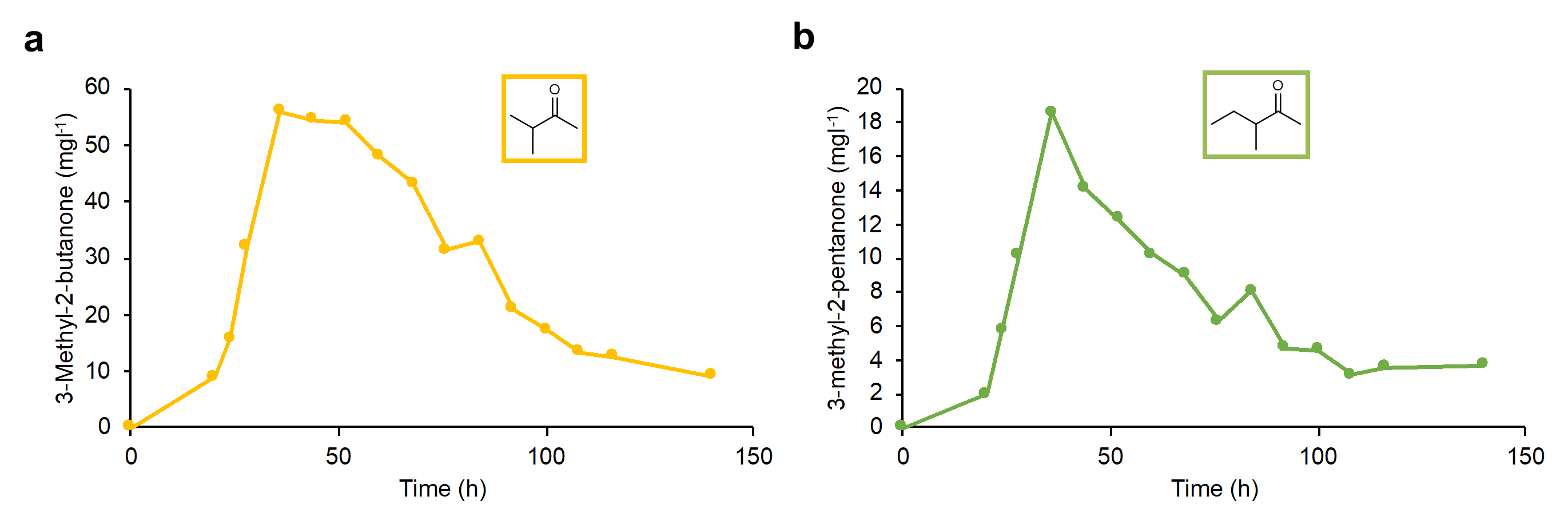
**

**Supplementary Fig. 10** **Short-chain ketone production in a bioreactor.** ALB189 was grown in a 2 L bioreactor containing 700 ml of Medium 042 at 30°C. Samples were taken from the bioreactor at different time points and production of 3-methyl-2-butanone (**a**) and 3-methyl-2-pentanone (**b**) were measured by LC-MS.


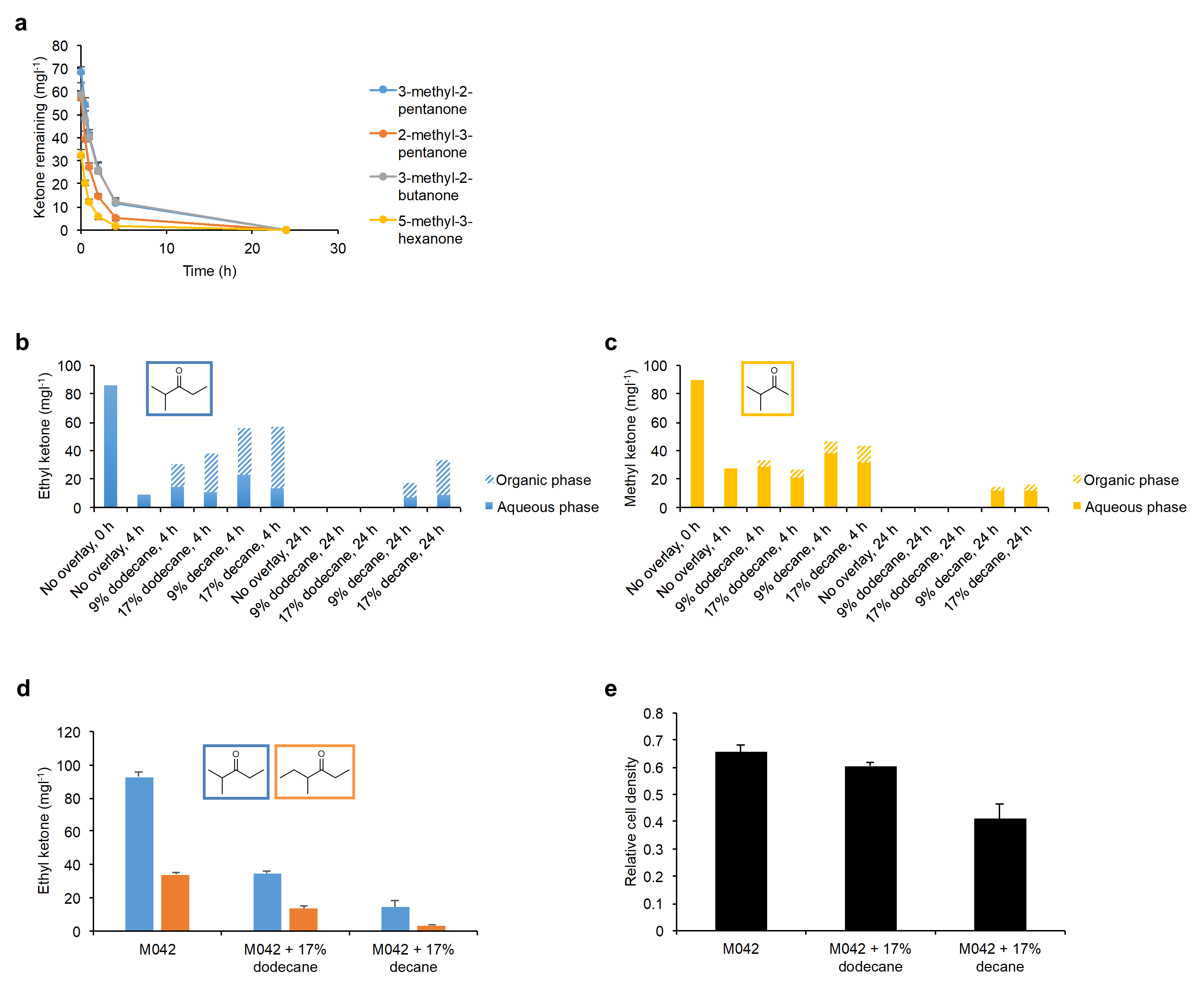


**Supplementary Fig. 11 Ketone evaporation and ketone production in Medium 042 with organic solvent overlay.** **a** 4 different short-chain ketones were added into Medium 042 and incubated at 30°C without cells. Samples were taken at different time points (0.5 h, 1 h, 2 h, 4 h, and 24 h) and the remaining amounts of ketones were quantified by LC-TOF-MS. Error bars are the S.D. from three independent experiments. 2-Methyl-3-pentanone (**b**) and 3-methyl-2-butanone (**c**) were added into Medium 042 in the presence of dodecane (9% or 17%) or decane (9% or 17%) and incubated at 30°C without cells. Samples were taken at different time points (4 h and 24 h) and the remaining amounts of ketones in the aqueous phase were quantified by LC-TOF-MS. GC-FID was used to quantify the remaining amounts of ketones in the organic phase. **d** ALB188 was cultured in M042 in the absence or the presence of dodecane (17%) or decane (17%) for 5 days at 30°C and ethyl ketone production was measured by LC-TOF-MS. 2-Methyl-3-pentanone and 4-methyl-3-hexanone are shown in blue and orange, respectively. Error bars are the S.D. from three independent experiments. 4-methyl-3-hexanone was quantified as 5-methyl-3-hexanone equivalent because 4-methyl-3-hexanone is not commercially available. **e** Relative cell density of ALB188 grown in M042 in the absence or the presence of dodecane (17%) or decane (17%) for 5 days were estimated by the Bradford assay. Error bars are the S.D. from three independent experiments.


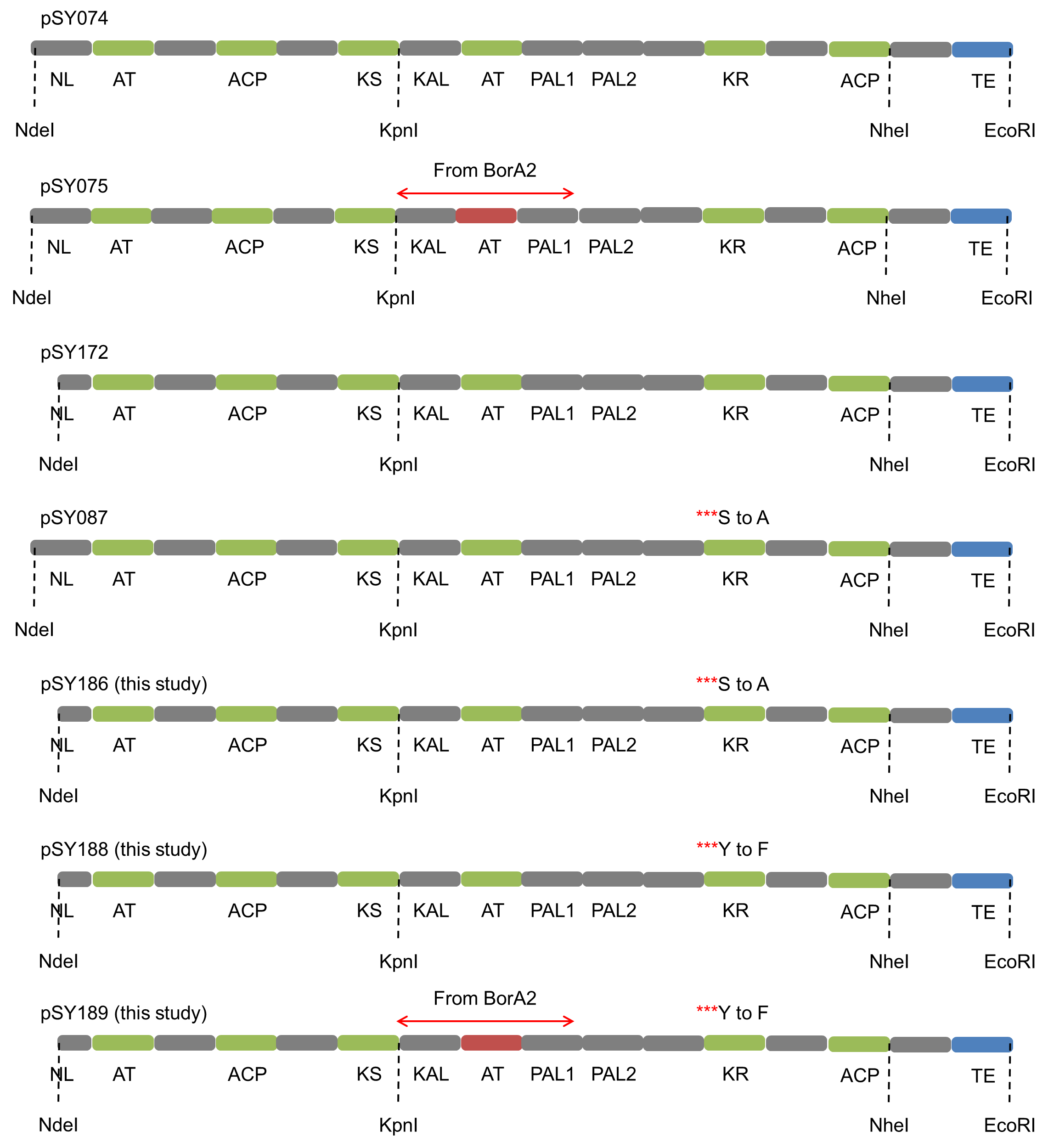


**Supplementary Fig. 12** **Plasmid construction.** Restriction enzymes sites and gene features in pSY074, pSY075, pSY172, pSY087, pSY186, pSY188, and pSY189 were shown.

**Supplementary Table 1.** **Promoters used in this study.**

| Promoter | Promoter sequence |
| --- | --- |
| gapdhp (EL) | GCTGCTCCTTCGGTCGGACGTGCGTCTACGGGCACCTTACCGCAGCCGTCGGCTGTGCGACACGGACGGATCGGGCGAACTGGCCGATGCTGGGAGAAGCGCGCTGCTGTACGGCGCGCACCGGGTGCGGAGCCCCTCGGCGAGCGGTGTGAAACTTCTGTGAATGGCCTGTTCGGTTGCTTTTTTTATACGGCTGCCAGATAAGGCTTGCAGCATCTGGGCGGCTACCGCTATGATCGGGGCGTTCCTGCAATTCTTAGTGCGAGTATCTGAAAGGGGATACGC |
| kasOp* | TGTTCACATTCGAACGGTCTCTGCTTTGACAACATGCTGTGCGGTGTTGTAAAGTCGTGGCCAGGAGAATACGACAGCGTGCAGGACTGGGGGAGTT |
| ermEp* | GTGCACGCGGTCGATCTTGACGGCTGGCGAGAGGTGCGGGGAGGATCTGACCGACGCGGTCCACACGTGGCACCGCGATGCTGTTGTGGGCACAATCGTGCCGGTTGGTAGGATCCAGCG |

**Supplementary Table 2.** **Primers used in genomic PCR experiments (see also Supplementary Fig. 2).**

| Primer name | PCR products | Primer sequence |
| --- | --- | --- |
| SY398 (Forward primer) | Amplify 1474 bp fragment (kasOp*), Amplify 1497 bp fragment (ermEp*), Amplify 1662 bp fragment (gapdhp), Amplify 1899 bp fragment (ALB33 and ALB37) | CACTCGACAAGTTCGTTGCGGAA |
| SY357 (Forward primer) | Amplify 1302 bp fragment | GGCGGTTCCGCTCTCCGTA |
| SY333 (Reverse primer) | Amplify 1474-1899 bp fragment with SY398, Amplify 1302 bp fragment with SY357 | GTAGCCTGCGCTTGGCTCCA |
| SY67 (Forward primer) | Amplify 2381 bp fragment | TGGACTCCGCACCGGTTTTCG |
| SY513 (Reverse primer) | Amplify 2381 bp fragment | AGAACCCAACCACAACGGGC |
| SY70 (Forward primer) | Amplify 2477 bp fragment (ethyl ketone PKS), Amplify 2408 bp fragment (methyl ketone PKS) | AGCGATGCGCGTCGTCTGG |
| SY514 (Reverse primer) | Amplify 2477 bp fragment (ethyl ketone PKS), Amplify 2408 bp fragment (methyl ketone PKS) | GTAACCATCCACAGCGGTGCA |
| SY73 (Forward primer) | Amplify 2709 bp fragment | GTTACCGCACCGCCTTTAAACG |
| SY332 (Reverse primer) | Amplify 2709 bp fragment | AGCTGTGCTTCCTGTTCGGCT |

**Supplementary Table 3. Expected size of PCR fragments from each construct in genomic PCR analysis (see also Supplementary Fig. 2).**

| ALB33 | 1899 bp, 2381 bp, 2477 bp, 2709 bp |
| --- | --- |
| ALB37 | 1899 bp, 2381 bp, 2408 bp, 2709 bp |
| ALB179 | 1474 bp, 2381 bp, 2477 bp, 2709 bp |
| ALB180 | 1474 bp, 2381 bp, 2408 bp, 2709 bp |
| ALB186 | 1662 bp, 2381 bp, 2477 bp, 2709 bp |
| ALB188 | 1662 bp, 2381 bp, 2477 bp, 2709 bp |
| ALB189 | 1662 bp, 2381 bp, 2408 bp, 2709 bp |
| ALB190 | 1302 bp, 2381 bp, 2477 bp, 2709 bp |
| ALB191 | 1302 bp, 2381 bp, 2408 bp, 2709 bp |
| ALB197 | 1497 bp, 2381 bp, 2477 bp, 2709 bp |
| ALB198 | 1497 bp, 2381 bp, 2408 bp, 2709 bp |
| AUR188 | 1662 bp, 2381 bp, 2477 bp, 2709 bp |
| AUR189 | 1662 bp, 2381 bp, 2408 bp, 2709 bp |
| COE188 | 1662 bp, 2381 bp, 2477 bp, 2709 bp |
| COE189 | 1662 bp, 2381 bp, 2408 bp, 2709 bp |
| VEN188 | 1662 bp, 2381 bp, 2477 bp, 2709 bp |
| VEN189 | 1662 bp, 2381 bp, 2408 bp, 2709 bp |

**Supplementary Table 4. Plasmids and strains used in this study**

| Name | Characteristic(s) | Source/reference |
| --- | --- | --- |
| Plasmids |  |  |
| p21 (JPUB_009893) | mCherry gene under gapdh promoter from *E. lenta*, ΦC31 attP and integrase, apramycin resistance | This study |
| p33 (JPUB_010344) | 2-Methyl-3-hydroxy acid PKS gene under gapdh promoter from *E. lenta*, ΦC31 attP and integrase, apramycin resistance | This study |
| p37 (JPUB_010346) | 3-Hydroxy acid PKS gene under gapdh promoter from *E. lenta*, ΦC31 attP and integrase, apramycin resistance | This study |
| pSY179 (JPUB_009542) | Ethyl ketone PKS (Y to F) gene under kasO* promoter, ΦC31 attP and integrase, apramycin resistance | This study |
| pSY180 (JPUB_009544) | Methyl ketone PKS gene (Y to F) under kasO* promoter, ΦC31 attP and integrase, apramycin resistance | This study |
| pSY186 (JPUB_010697) | Ethyl ketone PKS gene (S to A) under gapdh promoter from *E. lenta*, ΦC31 attP and integrase, apramycin resistance | This study |
| pSY188 (JPUB_009546) | Ethyl ketone PKS gene (Y to F) under gapdh promoter from *E. lenta*, ΦC31 attP and integrase, apramycin resistance | This study |
| pSY189 (JPUB_009548) | Methyl ketone PKS gene (Y to F) under gapdh promoter from *E. lenta*, ΦC31 attP and integrase, apramycin resistance | This study |
| pSY190 (JPUB_009887) | Ethyl ketone PKS gene (Y to F) under gapdh promoter from *E. lenta*, VWB attP and integrase, apramycin resistance | This study |
| pSY191 (JPUB_009895) | Methyl ketone PKS gene (Y to F) under gapdh promoter from *E. lenta*, VWB attP and integrase, apramycin resistance | This study |
| pSY197 (JPUB_009889) | Ethyl ketone PKS gene (Y to F) under ermE* promoter, ΦC31 attP and integrase, apramycin resistance | This study |
| pSY198 (JPUB_009891) | Methyl ketone PKS gene (Y to F) under ermE* promoter, ΦC31 attP and integrase, apramycin resistance | This study |
| Strains |  |  |
| *E. coli* |  |  |
| DH10B | Cloning host |  |
| ET12567/pUZ8002 | Donor strain for conjugation between *E. coli* and *Streptomyces* | ^1^ |
| *S. albus* |  |  |
| J1074 | Wild type host strain | ^2^ |
| ALB21 (JPUB_009902) | Derivative of J1074 containing p21 integrated at ΦC31 *attB* chromosomal site | This study |
| ALB33 (JPUB_010343) | Derivative of J1074 containing p33 integrated at ΦC31 *attB* chromosomal site | This study |
| ALB37 (JPUB_010346) | Derivative of J1074 containing p37 integrated at ΦC31 *attB* chromosomal site | This study |
| ALB179 (JPUB_009903) | Derivative of J1074 containing pSY179 integrated at ΦC31 *attB* chromosomal site | This study |
| ALB180 (JPUB_009904) | Derivative of J1074 containing pSY180 at ΦC31 *attB* chromosomal site | This study |
| ALB186 (JPUB_010698) | Derivative of J1074 containing pSY186 integrated by the at ΦC31 *attB* chromosomal site | This study |
| ALB188 (JPUB_009905) | Derivative of J1074 containing pSY188 integrated by the at ΦC31 *attB* chromosomal site | This study |
| ALB189 (JPUB_009906) | Derivative of J1074 containing pSY189 integrated at ΦC31 *attB* chromosomal site | This study |
| ALB190 (JPUB_009909) | Derivative of J1074 containing pSY190 integrated at VWB *attB* chromosomal siteintegrase-mediated recombination | This study |
| ALB191 (JPUB_009910) | Derivative of J1074 containing pSY191 integrated at VWB *attB* chromosomal site | This study |
| ALB197 (JPUB_009907) | Derivative of J1074 containing pSY197 integrated at ΦC31 *attB* chromosomal site | This study |
| ALB198 (JPUB_009908) | Derivative of J1074 containing pSY198 integrated at ΦC31 *attB* chromosomal site | This study |
| *S. aureofaciens* |  |  |
| Tü117 | Wild type host strain | ^3^ |
| AUR21 (JPUB_009899) | Derivative of Tü117 containing p21 integrated at ΦC31 *attB* chromosomal site | This study |
| AUR188 (JPUB_009900) | Derivative of Tü117 containing pSY188 integrated at ΦC31 *attB* chromosomal site | This study |
| AUR189 (JPUB_009901) | Derivative of Tü117 containing pSY189 integrated at ΦC31 *attB* chromosomal site | This study |
| *S. coelicolor* |  |  |
| A3(2) | Wild type host strain | ^4^ |
| COE21 (JPUB_009911) | Derivative of A3(2) containing p21 integrated at ΦC31 *attB* chromosomal site | This study |
| COE188 (JPUB_009912) | Derivative of A3(2) containing pSY188 integrated at ΦC31 *attB* chromosomal site | This study |
| COE189 (JPUB_009913) | Derivative of A3(2) containing pSY189 integrated at ΦC31 *attB* chromosomal site | This study |
| *S. venezuelae* |  |  |
| ATCC 10712 | Wild type host strain | ^5^ |
| VEN21 (JPUB_009896) | Derivative of ATCC 10712 containing p21 integrated at ΦC31 *attB* chromosomal site | This study |
| VEN188 (JPUB_009897) | Derivative of ATCC 10712 containing pSY188 integrated at ΦC31 *attB* chromosomal site | This study |
| VEN189 (JPUB_009898) | Derivative of ATCC 10712 containing pSY189 integrated at ΦC31 *attB* chromosomal site | This study |

**Supplementary Table 5. Media used in this study.**

| Medium | Composition | Source/reference |
| --- | --- | --- |
| 2x YT |  | Sigma-Aldrich, Y2377 |
| TSB |  | EMD Millipore, 1.00525.5007 |
| Medium 042 | Glucose (10 g⋅l^-1^), Glycerol (10 g⋅l^-1^), Corn starch (10 g⋅l^-1^), Corn steep (2.5 g⋅l^-1^), Peptone (5 g⋅l^-1^), Yeast extract (2 g⋅l^-1^), NaCl (1 g⋅l^-1^), CaCO_3_ (3 g⋅l^-1^), pH 7.2 | ^6^ |
| Modified Medium 042 | Corn steep (2.5 g⋅l^-1^), Peptone (5 g⋅l^-1^), Yeast extract (2 g⋅l^-1^), NaCl (1 g⋅l^-1^), CaCO_3_ (3 g⋅l^-1^), pH 7.2 + Corn stover alkaline hydrolysate that contains Glucose (65 g⋅l^-1^), Xylose (22 g⋅l^-1^) | This study |
| Modified Medium 042 + Val | Valine (2.4 g⋅l^-1^), Corn steep (2.5 g⋅l^-1^), Peptone (5 g⋅l^-1^), Yeast extract (2 g⋅l^-1^), NaCl (1 g⋅l^-1^), CaCO_3_ (3 g⋅l^-1^), pH 7.2 + Corn stover alkaline hydrolysate that contains Glucose (65 g⋅l^-1^), Xylose (22 g⋅l^-1^) | This study |
| Modified Medium 042 + Ile | Isoleucine (2.6 g⋅l^-1^), Corn steep (2.5 g⋅l^-1^), Peptone (5 g⋅l^-1^), Yeast extract (2 g⋅l^-1^), NaCl (1 g⋅l^-1^), CaCO_3_ (3 g⋅l^-1^), pH 7.2 + Corn stover alkaline hydrolysate that contains Glucose (65 g⋅l^-1^), Xylose (22 g⋅l^-1^) | This study |
| Modified Medium 042 + Thr | Threonine (2.4 g⋅l^-1^), Corn steep (2.5 g⋅l^-1^), Peptone (5 g⋅l^-1^), Yeast extract (2 g⋅l^-1^), NaCl (1 g⋅l^-1^), CaCO_3_ (3 g⋅l^-1^), pH 7.2 + Corn stover alkaline hydrolysate that contains Glucose (65 g⋅l^-1^), Xylose (22 g⋅l^-1^) | This study |
| Modified Medium 042 + 3 amino acids | Valine (2.4 g⋅l^-1^), Isoleucine (2.6 g⋅l^-1^), Threonine (2.4 g⋅l^-1^), Corn steep (2.5 g⋅l^-1^), Peptone (5 g⋅l^-1^), Yeast extract (2 g⋅l^-1^), NaCl (1 g⋅l^-1^), CaCO_3_ (3 g⋅l^-1^), pH 7.2 + Corn stover alkaline hydrolysate that contains Glucose (65 g⋅l^-1^), Xylose (22 g⋅l^-1^) | This study |
| ISP-2 | Glucose (4 g⋅l^-1^), Yeast extract (4 g⋅l^-1^), Malt extract (10 g⋅l^-1^), pH 7.2 | ^6^ |
| MYM | Maltose (4 g⋅l^-1^), Yeast extract (4 g⋅l^-1^), Malt extract (10 g⋅l^-1^), pH 7.2 | ^7^ |
| B | Glucose (20 g⋅l^-1^), Soluble starch (5 g⋅l^-1^), Peptone (2 g⋅l^-1^), Yeast extract (2 g⋅l^-1^), Soy flour (10 g⋅l^-1^), K_2_HPO_4_ (0.5 g⋅l^-1^), Mg_2_SO_4_⋅7H^2^O (0.5 g⋅l^-1^), NaCl (4 g⋅l^-1^), CaCO_3_ (2 g⋅l^-1^), pH 7.8 | ^6^ |

**Supplementary Table 6.** **A comparison of fuel properties of isooctane, common biofuels, and ketones produced by engineered *S. albus* strains.**

|  | Isooctane | Ethanol | 1-Butanol | 2-Methyl-3-pentanone | 3-Methyl-2-butanone |
| --- | --- | --- | --- | --- | --- |
| CAS | 540-84-1 | 64-17-5 | 71-36-3 | 565-69-5 | 563-80-4 |
| Formula | C_8_H_18_ | C_2_H_6_O | C_4_H_10_O | C_6_H_12_O | C_5_H_10_O |
| Molecular weight | 114.23 | 46.07 | 74.12 | 100.16 | 86.13 |
| Research Octane number (RON) | 100  (by definition) | 109^a^ | 98^a^ | 100  (this study) | 109^a^ |
| Motor Octane Number (MON) | 100  (by definition) | 90^a^ | 85^a^ | 100  (this study) | 102^a^ |
| Sensitivity (RON-MON) | 0 | 19 | 13 | 0 | 7 |
| Anti-Knock Index = AKI [(RON+MON)/2] | 100 | 99.5 | 91.5 | 100 | 105.5 |
| Specific energy [MJ⋅kg^-1^] | 44.2^b^ | 27.2^b^ | 33.2^b^ | 34.7^b^ | 33.9^b^ |
| Density [kg⋅m^-3^] | 692 (25 °C)^c^ | 789 (25 °C)^c^ | 810 (25 °C)^c^ | 811 (25 °C)^c^ | 805 (25 °C)^c^ |
| Energy density [MJ⋅m^-3^] | 30.6 | 21.5 | 26.9 | 28.1 | 27.3 |
| Boiling point [°C] | 98-99^c^ | 78^c^ | 116-118^c^ | 113^c^ | 94-95^c^ |
| Melting point [°C] | -107^c^ | -114^c^ | -90^c^ | - | -92^c^ |
| Flash point [°C] | -7^c^ | 13^c^ | 35^c^ | 11^c^ | -1^c^ |
| Auto ignition temperature [°C] | 396.1^c^ | 361.7^c^ | 342.8^c^ | 440  (this study) | 415  (this study) |
| Vapor pressure [mmHg] | 41 (21 °C)^c^ | 44.6 (20 °C)^c^ | 4 (20 °C)^c^ | - | 52.5 (25 °C)^e^ |
| Water solubility [g⋅l^-1^] | Immiscible | 1000.0 (25 °C)^d^ | 63.2 (25 °C)^d^ | 15.5 (25 °C)^d^ | 52.4 (20 °C)^e^ |

a: reported by McCormick et al^8^.

b: each specific energy was calculated based on average bond enthalpies at 25 °C.

c: the data were collected from Sigma-Aldrich and Alfa Aesar.

d: the data were collected from Human Metabolome Data Base (http://www.hmdb.ca/metabolites/HMDB0005846)

e: the data were collected from Hazardous Substances Data Bank (https://toxnet.nlm.nih.gov/cgi-bin/sis/search/r?dbs+hsdb:@term+@rn+@rel+563-80-4)

**Supplementary Table 7.** **Primers used to construct plasmids.**

| Primer | Primer sequence | Note |
| --- | --- | --- |
| SY345 | ACTAGTACTCCAGCCCGACCCGA | Insert SpeI site to *Streptomyces* integration vector (apramycin^R^, VWB)^7^ |
| SY346 | CTCGTTCGTTCACACGTTGCAG | Insert SpeI site to *Streptomyces* integration vector (apramycin^R^, VWB)^7^ |
| SY479 | TTGCGGCTGCGAACGCATTTC | Inactivate the KR domain (Y to F) |
| SY480 | ACGCGCCGTGGTCGCCGGA | Inactivate the KR domain (Y to F) |
| phiC31_int_F | AAATTTGCATGCCTGACGCCGTTGGATAC | Insert ΦC31 attP/int into *Streptomyces* integration vector (apramycin^R^, VWB)^7^ using SphI and SpeI |
| phiC31_int_R | AAATTTACTAGTCTGCTGCAACGTGTGAACGAACG | Insert ΦC31 attP/int into *Streptomyces* integration vector (apramycin^R^, VWB)^7^ using SphI and SpeI |
| SY343 | GCGCGACTAGTTGTTCACATTCGAACGGTCTCTGCTTTGACAACATGCTGTGCGGTGTTGTAAAGTCGTG | Insert kasOp* into *Streptomyces* integration vector (apramycin^R^, VWB)^7^ using SpeI and NdeI |
| SY344 | CGCGCCATATGAACTCCCCCAGTCCTGCACGCTGTCGTATTCTCCTGGCCACGACTTTACAACACCGCACA | Insert kasOp* into *Streptomyces* integration vector (apramycin^R^, VWB)^7^ using SpeI and NdeI |
| SY347 | GCGCGACTAGTGTGCACGCGGTCGATCTTGA | Insert ermEp* into *Streptomyces* integration vector (apramycin^R^, VWB)^7^ using SpeI and NdeI |
| SY348 | CGCGCCATATGCGCTGGATCCTACCAACCGGCACGA | Insert ermEp* into *Streptomyces* integration vector (apramycin^R^, VWB)^7^ using SpeI and NdeI |
| SY349 | GCGCGACTAGTGCTGCTCCTTCGGTCGGAC | Insert gapdhp (EL) into *Streptomyces* integration vector (apramycin^R^, VWB)^7^ using SpeI and NdeI |
| SY350 | CGCGCCATATGGCGTATCCCCTTTCAGATACTC | Insert gapdhp (EL) into *Streptomyces* integration vector (apramycin^R^, VWB)^7^ using SpeI and NdeI |

**Supplementary References**

1. Kieser, T., Bibb, M.J., Buttner, M.J., Chater, K.F. & Hopwood, D.A. Practical streptomyces genetics. *The John Innes Foundation* (2000).

2. Zaburannyi, N., Rabyk, M., Ostash, B., Fedorenko, V. & Luzhetskyy, A. Insights into naturally minimised Streptomyces albus J1074 genome. *BMC Genomics* **15**, 97 (2014).

3. Bihlmaier, C. et al. Biosynthetic gene cluster for the polyenoyltetramic acid alpha-lipomycin. *Antimicrob Agents Chemother* **50**, 2113-2121 (2006).

4. Bentley, S.D. et al. Complete genome sequence of the model actinomycete Streptomyces coelicolor A3(2). *Nature* **417**, 141-147 (2002).

5. Pullan, S.T., Chandra, G., Bibb, M.J. & Merrick, M. Genome-wide analysis of the role of GlnR in Streptomyces venezuelae provides new insights into global nitrogen regulation in actinomycetes. *BMC Genomics* **12**, 175 (2011).

6. Rateb, M.E. et al. Medium optimization of Streptomyces sp. 17944 for tirandamycin B production and isolation and structural elucidation of tirandamycins H, I and J. *J Antibiot (Tokyo)* **67**, 127-132 (2014).

7. Phelan, R.M. et al. Development of Next Generation Synthetic Biology Tools for Use in Streptomyces venezuelae. *ACS Synth Biol* **6**, 159-166 (2017).

8. McCormick, R.L. et al. Selection Criteria and Screening of Potential Biomass-DerivedStreams as Fuel Blendstocks for Ad vanced Spark-Ignition Engines. *SAE Int J Fuels Lubr* **10**, 442-460 (2017).
