# Supplementary material for: Short-chain ketone production by engineered polyketide synthases in Streptomyces albus
Source: Nat Commun. 2018 Nov 1;9:4569. doi: 10.1038/s41467-018-07040-0 (PMC6212451; doi:10.1038/s41467-018-07040-0)
Supplement: Supplementary file 3 — Description of Additional Supplementary Files [file 41467_2018_7040_MOESM3_ESM.pdf]

Description of Additional Supplementary Files

Supplementary Data 1
Description: Amino acid sequences of natural and synthetic enzymes used in this study (EryAIII = blue, LipPks1 = green, BorA2 = magenta, others = black, mutation = orange bold).
